# AGE-RELATED DIFFERENCES IN IMMUNO-HEMATOLOGIC PROFILES AND THEIR ASSOCIATION WITH ALL-CAUSE MORTALITY

**DOI:** 10.1093/geroni/igz038.388

**Published:** 2019-11-08

**Authors:** Jarrod E Dalton, David A Zidar, Nikolas I Krieger, Adam T Perzynski, Douglas D Gunzler

**Affiliations:** 1 Cleveland Clinic Lerner College of Medicine at Case Western Reserve University, Cleveland, Ohio, United States; 2 University Hospitals Cleveland Medical Center, Cleveland, Ohio, United States; 3 Cleveland Clinic, Cleveland, Ohio, United States; 4 Case Western Reserve University at MetroHealth, Cleveland, Ohio, United States

## Abstract

Immuno-hematologic function (IHF) is increasingly being recognized as a central component of health status in older age. In this study, we sought to identify homogeneous IHF profiles regarding their relationship to all-cause mortality. We then studied the distribution of these profiles among individuals over age 65. We used data on 30,828 NHANES participants, including 10 baseline complete blood count with differential components [e.g., lymphocytes, leukocytes, red cell distribution width (RDW)] and all-cause mortality. We used latent profile analysis (LPA) to simultaneously optimize intra-cluster homogeneity on CBC components and inter-cluster survival differences. LPA (using MPlus 8.2) allowed for the empirical comparison of different solutions based on goodness-of-fit criteria. After LPA model convergence, a 9-class solution balanced goodness-of-fit criteria and interpretability of the resulting classes. The largest 3 classes accounted for 83.7% of the sample, with classes 1, 2 and 3 comprising 32.1%, 28.6% and 23.6%. Class 2 had lower lymphocytes, monocytes, neutrophils and platelets relative to classes 1 and 3. Survival rates were different between classes 1 and 2 (Cox model hazard ratio, HR=0.85; P=0.012) and 2 vs 3 (HR=1.18; P=0.001). The remaining 6 classes, which generally shared in common characteristics of higher RDW and lower hemoglobin, also were involved with significant survival differences. Multinomial logistic regression revealed that, among the subset of 7,173 participants over 65, older age was significantly associated with membership in class 1 relative to classes 2 (P<0.001) and 3 (P<0.001). These results point toward the possibility of developing immune marker profile indicative of accelerated aging.

